# PNcsp+: A Periodic Number-Based Crystal Structure Prediction Method Enhanced by Machine Learning

**DOI:** 10.1021/acs.jctc.6c00044

**Published:** 2026-03-19

**Authors:** Cem Oran, Riccarda Caputo, Pierre Villars, Adem Tekin

**Affiliations:** † 52971 Informatics Institute, Istanbul Technical University, Istanbul 34469, Türkiye; ‡ Computational Materials Informatics - CMI, Rome 00157, Italy; § MPDS-Villars, Vitznau 6354, Switzerland; ∥ Tübi̇tak Research Institute for Fundamental Sciences, Gebze 41470, Türkiye

## Abstract

Crystal structure prediction (CSP) is central to materials discovery, yet its efficiency and interpretability remain limited by the vast configurational space and reliance on costly local optimizations. Although template-based and machine-learning (ML) approaches have improved exploration, many approaches still require large data sets, complex similarity metrics, or opaque generative pipelines. In this work, we introduce PNcsp+, an enhanced and chemically interpretable CSP framework that uses the Mendeleev Periodic Number (PN) as a transparent descriptor of elemental similarity. PNcsp+ expands the original implementation through a larger prototype library, an improved data management strategy, and ML-assisted prototype scoring by combining cutting-edge neural network models such as MACE, M3GNet, and ALIGNN-FF. Despite its simplicity, PNcsp+ reaches state-of-the-art performance. In evaluations on the CSPBench data set—a curated set of 180 benchmark crystal structures for assessing CSP methods—our approach surpasses alternative methods by achieving 86.1% space group accuracy and 85.0% structure matching accuracy within the Top-5 predictions, all without structure relaxations. Moreover, our case study on several hybrid systems, including ammonium and methylammonium cations, demonstrated that molecular components emerge autonomously in the predicted lattices, guided solely by PN-derived similarity relationships. Overall, PNcsp+ shows that fundamental periodic trends, combined with targeted ML-based evaluation, offer an efficient, scalable, and interpretable CSP framework, enabling accelerated discovery across both inorganic and hybrid chemical spaces.

## Graphical abstract

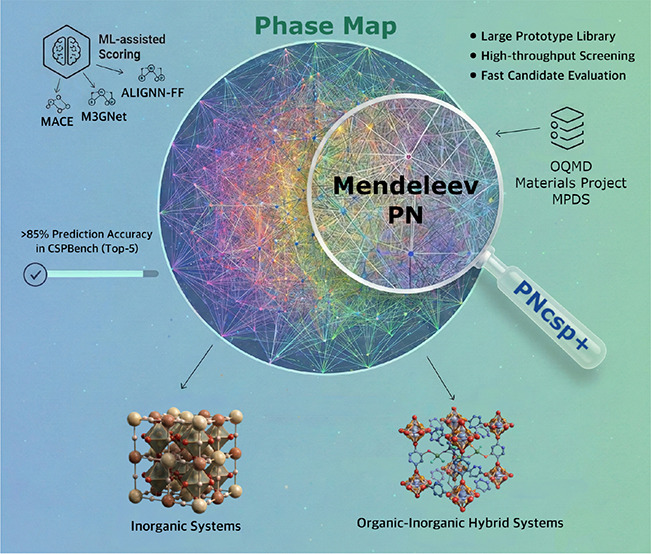

## Introduction

Recent advances in theoretical and computational methodologies have made it possible to systematically investigate an increasingly broad class of compounds. The primary motivation behind these efforts is the discovery of novel materials with properties precisely tailored for targeted applications, enabling technological breakthroughs. In this regard, crystal structure prediction (CSP) constitutes a central research area in materials science, aiming at identifying stable solid-state phases whose crystal structure determination might be challenging to resolve experimentally. Predicting crystal structures directly through ab initio geometry optimization using widely employed density functional theory (DFT) packages such as VASP^1^ and CASTEP^2^ can deliver highly accurate results; however, because they rely on local optimization schemes, the final structures are strongly dependent on the quality of the initial guess. Moreover, the computational cost of DFT calculations makes the exploration of configuration space impractical for complex systems. The integration of machine learning (ML) algorithms with high-performance computing infrastructures has significantly enhanced this process, allowing more accurate and efficient analyses of large structural and compositional spaces. But completely relying on “black-box” ML models has its own drawbacks, such as limited interpretability, which is the main factor that hinders the extraction of meaningful chemical insight.

A number of studies^3–6^ have proposed approaches that incorporate the empirical chemical knowledge into the search process. For phases with unknown crystal structure representation, the most effective strategy is the exploration of phase space by using a set of prototype structures and evaluating the chemical similarities between constituent elements. A recent study^7^ introduced a comprehensive benchmark data set consisting of 180 test systems (CSPBench set) and evaluated the performance of 13 CSP programs. The results demonstrated that template-based approaches achieve the most consistent and reliable predictive performance. Complementing this, another recent study^8^ evaluated the performance of template-based method, TCSP, against two of the most prominent alternatives, CSPML^9^ and EquiCSP^10^ through the same CSPBench data set.^7^ This study provided a unified perspective on the strengths and limitations of both template-driven and ML-based methods in CSP.

CSPML represents a template-based algorithm that employs metric learning to guide the selection of prototype structures whose compositions are chemically “replaceable” with the target composition. Once promising templates are selected, CSPML performs elemental substitution and then carries out local relaxation on the substituted structures. This design situates CSPML as an ML extension of the traditional template-driven discovery pipelines. In contrast, EquiCSP is an equivariant diffusion-based generative model for the CSP that directly builds crystal symmetries into its design. It ensures that the generated structures always respect the fundamental symmetry constraints—like how atoms repeat across the lattice and how the crystal translates periodically. By doing so, EquiCSP produces more realistic crystal candidates and learns faster than earlier diffusion-based methods.

In parallel, TCSP 2.0 is an enhanced template-based CSP approach that upgrades its predecessor by replacing traditional oxidation state assignment with the deep learning BERTOS^11^ model, and by introducing element embedding distance metrics and a majority-voting scheme for the space group selection. These innovations—together with an expanded template library and CHGNet^12^-based structure relaxation—enable TCSP 2.0 to achieve substantially higher accuracy in both structure matching and space group prediction compared to the previous CSP approaches.

In all these approaches, whether template-based or ML-driven, the ability to distinguish between chemically similar and dissimilar systems remains a central challenge. In both Wei et al.^13^ and Wang et al.^6^ such chemical similarity metrics were constructed through complex analyses of large data sets and intricate structural correlations. Recently, we have also developed a template-based CSP framework, PNcsp^14^ which employs a similarity metric based on the Mendeleev Periodic Number (PN).^15^ In its current enhanced implementation, PNcsp+, the method integrates state-of-the-art ML models dedicated to prototype evaluation and an improved data-reduction pipeline. Moreover, the prototype inventory has been substantially expanded, and the overall data-management strategy has been improved.

In contrast to alternative approaches, PNcsp+ captures similarity relationships in a remarkably simple and interpretable manner. This conceptual simplicity constitutes the principal advantage of our method over existing alternatives. Whereas many existing methods require generating large numbers of potentially “similar” prototype systems and subsequently applying atomic substitutions, the PN-based approach drastically reduces the number of candidates that must be considered. Despite this streamlined process, PNcsp+ still surpasses alternative methods in accurately reproducing stable crystal structure configurations. Our framework was applied to 180 test systems included in the CSPBench set, and its performance was compared with the most successful CSP approaches, such as TCSP 2.0, CSPML, and EquiCSP. Furthermore, the method was tested on a set of challenging hybrid organic–inorganic compounds containing ammonium and methylammonium cations. Remarkably, the molecular cations emerged naturally within the predicted lattice frameworks based solely on PN-derived similarity relationships, highlighting the framework’s adaptability beyond purely inorganic chemistry.

## Methodology

### PN Concept and Overview of the PNcsp Approach

The Mendeleev PN is an ordering index assigned to chemical elements such that chemically similar elements appear consecutively. Although the atomic number is fundamental, it does not fully satisfy this objective, as it progresses period by period rather than grouping elements according to chemical similarity. The first formally defined periodic enumeration of this kind was introduced by Pettifor in 1984 ^16^ through a phenomenological optimization aimed at separating binary AB compounds into distinct structure types. This ordering effectively traverses the periodic table group by group, leading to characteristic rearrangements, such as positioning Eu and Yb after Ca and Pb after Ga.^17^

The Mendeleev PN enumeration, developed by Pierre Villars and coworkers^18^ and employed in this study (hereafter referred to simply as PN), is more directly derived from periodic trends. In this representation, elements are ordered group-wise from top to bottom and sequentially from left to right across the periodic table. Compared with conventional layouts, H is placed above the halogen group, while Be and Mg are positioned above group 12 (Zn, Cd, Hg) to better reflect chemical similarity.

In fact, the PN incorporates the principal quantum number (the period of the Periodic Table) and the valence shell configuration, while moving down along the group. In this scheme, elements with similar PN values exhibit closely related chemical properties. A detailed description of the systematic enumeration of all 118 chemical elements within the PN space is provided in the (Supporting Information SI).

By using the PN of the constituent elements of chemical systems of any order, we can draw a phase map, the PN-representation of the phase space, and identify crystal structure similarities and physical property trends in a straightforward way. Recently, we have demonstrated that the PN representation of the phase map of binary and higher-order chemical systems clearly pointed out the existence of forming, where stable phases are observed, and nonforming regions, where no known phases form except a small number of accepted violations.^15^ This representation, extendable from binary to higher-order systems, not only delineates stability domains with clarity but also reveals chemical trends and structural similarities across neighboring systems, including correlations in enthalpy of formation, mechanical properties, and prototypes.

The PN as a similarity metric was first implemented in our initial algorithm of PNcsp^14^ where it served as a similarity metric for predicting the crystal structures of binary phases identified in the binary phase diagrams^19^ but whose crystal structures are not yet determined experimentally. Unlike conventional approaches, the PNcsp methodology exploits this ordering to formalize the notion of chemical interchangeability. Specifically, structural prototypes are generated by systematically replacing atoms within known crystalline motifs with alternative elements whose PN values indicate comparable elemental properties. Consequently, structural similarity assessment is (i) simple, relying on a single descriptor, and (ii) interpretable, being rooted in an explicit chemical rationale rather than in latent learned representations. This quantitative basis for substitution represents the central conceptual distinction between our approach and those reported previously by other groups.

The exploration of substitution pathways proceeds in an iterative manner. The search begins with the first-order nearest neighbors in the PN representation of the phase map and gradually extends to higher orders (second, third, and so on), thereby constructing a controlled expansion of candidate systems. The distance metric in PN space is defined as the absolute difference between the PNs of the constituent elements, |*PN*_1_ – *PN*_2_|. To restrict the chemical space to the most likely stable phases, only the phases with negative formation enthalpy constitute the pool of neighbor phases of a target phase. Once the potential similar phases are identified, the corresponding structure types are considered for the elemental substitution process.

### Enhancements and New Functionalities in PNcsp+

As a major enhancement over the original implementation, PNcsp+ integrates an AI-based pre-evaluation module that markedly lowers the computational cost of traditional DFT-based screening and optimization steps. A review of the literature reveals that graph neural networks (GNNs)—owing to their natural ability to capture structure–property relationships in molecular and covalently bonded systems—are among the most widely adopted and accurate ML approaches for predicting the energies and, consequently, the thermodynamic stabilities of crystalline materials.^20^ Among these, MegNet^21^ and its extended variant M3GNet^22^ which accounts for three-body interactions, have been successfully employed as surrogates for DFT calculations. Moreover, alternative models with distinct architectures, such as MACE^23^ and ALIGNN-FF^24^ representing both equivariant and nonequivariant GNN designs, have also demonstrated high accuracy in crystal structure energy prediction tasks. Building upon these advances, the pre-evaluation module in PNcsp+ harnesses these state-of-the-art GNN models—specifically MACE, ALIGNN-FF, and M3GNet—to perform rapid single-point energy predictions for the generated structural prototypes.

Given that publicly available pretrained versions of these models have consistently demonstrated near-DFT accuracy in reproducing energies and forces across diverse materials data sets, they are incorporated directly into the PNcsp+ workflow without additional fine-tuning. During the final selection stage, the predictions from the three GNN models can be combined through an ensemble averaging strategy to improve robustness and mitigate model-specific biases. In this approach, the predicted energies from the selected models (all three or a subset thereof) are averaged to obtain a consensus estimate, thereby stabilizing the ranking of candidate structures.

Beyond single-point energy prediction, these models are also integrated into an optional structure relaxation pipeline, providing a scalable alternative to the DFT-based relaxation procedure employed in the original implementation. As an additional refinement aimed at reducing computational cost during the structure relaxation step, an optional redundant-prototype elimination process is incorporated through pymatgen’s StructureMatcher module prior to GNN-based assessment. This procedure effectively filters out structurally similar prototypes, ensuring that only unique candidates are passed forward for subsequent structure relaxation. This process enhances the diversity of the candidate pool, allowing the GNN models to focus on genuinely distinct structural configurations.

It is important to note that both structure relaxation and redundant-prototype elimination are optional features and were not employed in the benchmarking analysis presented in this work, as detailed in the following section. While structure relaxation can further improve ranking accuracy, albeit at significantly increased computational cost. Accordingly, PNcsp+ incorporates relaxation as an optional stage, allowing users to balance computational efficiency against predictive refinement. The structure ranking may be applied either by skipping relaxation for rapid screening or after relaxation for enhanced energetic refinement. This design provides methodological flexibility, enabling users to tailor the workflow according to available resources and accuracy requirements.

Significant enhancements have also been implemented in the data-source architecture and data-management strategy. The original PNcsp framework was primarily designed for equimolar binary systems, relying mainly on a locally generated data set. Its applicability to more complex, nonequimolar, and multicomponent systems has been verified only through limited preliminary tests. In PNcsp+, we extended the pool of phases comprising higher-order systems and any possible constitutions by adopting the Open Quantum Materials Database (OQMD)^25^ as the primary source. While numerous computational structure databases exist, OQMD offers distinct advantages owing to the sheer volume of its data—containing over 1.4 million ICSD-derived and hypothetical structures—and its focus on structural decorations. Rather than prioritizing curated properties of experimentally known compounds, OQMD systematically explores hypothetical chemical space by decorating common crystal prototypes (e.g., perovskites, Heusler alloys, and spinels) with a large variety of feasible elemental combinations.

Moreover, the extensive enumeration of stoichiometric variants enables smoother and more complete convex hull construction for thermodynamic stability analysis. For a candidate compound *A*_*x*_ *B*_*y*_ *C*_*z*_, OQMD typically includes many competing nearby phases, allowing more reliable evaluation of the energy above hull (*E*_hull_) against a nearly exhaustive set of competitors, thereby functioning as a large-scale prototype reference space. Furthermore, unlike databases primarily accessed through web APIs, OQMD’s qmpy framework supports full local deployment, facilitating integration into high-throughput CSP workflows. In PNcsp+, local deployment enabled offline querying with minimal latency and without API limitations, eliminating data-access bottlenecks present in earlier implementations and enabling efficient large-scale structural screening. This design makes OQMD particularly well-suited for template-based CSP.

In addition, complementary repositories have also been integrated, such as the Materials Project (MP)^26^ which is particularly strong in systems with organic components, and the Materials Platform for Data Science (MPDS)^19^ recognized as the world’s largest experimental materials database, fully curated by specialists in crystallography and materials science. Full access to the MPDS platform is reserved for registered users, whereas only a limited amount of content is publicly available. When activated, these auxiliary databases expand the search space and enhance the likelihood of identifying relevant and structurally analogous prototypes, further strengthening the robustness and flexibility of the PNcsp+ framework.

Figure 1 illustrates the PNcsp+’s multistep prediction process. Initially, the program generates candidate structures by assessing crystal structure type similarity within the PN representation of the phase map. These candidates are subsequently refined by a GNN-based evaluator module, either through single-point energy prediction or optional structure relaxation, leading to the final selection of the top-n results. No additional structural feature engineering or chemical rule encoding is required. Regarding hyperparameters and user-defined settings, the framework requires only a small number of choices: the number of nearest neighbors to include, the GNN model used for evaluation, and whether structure relaxation is enabled. Thus, the workflow does not involve extensive hyperparameter tuning or trial-and-error optimization.

**Figure 1:**
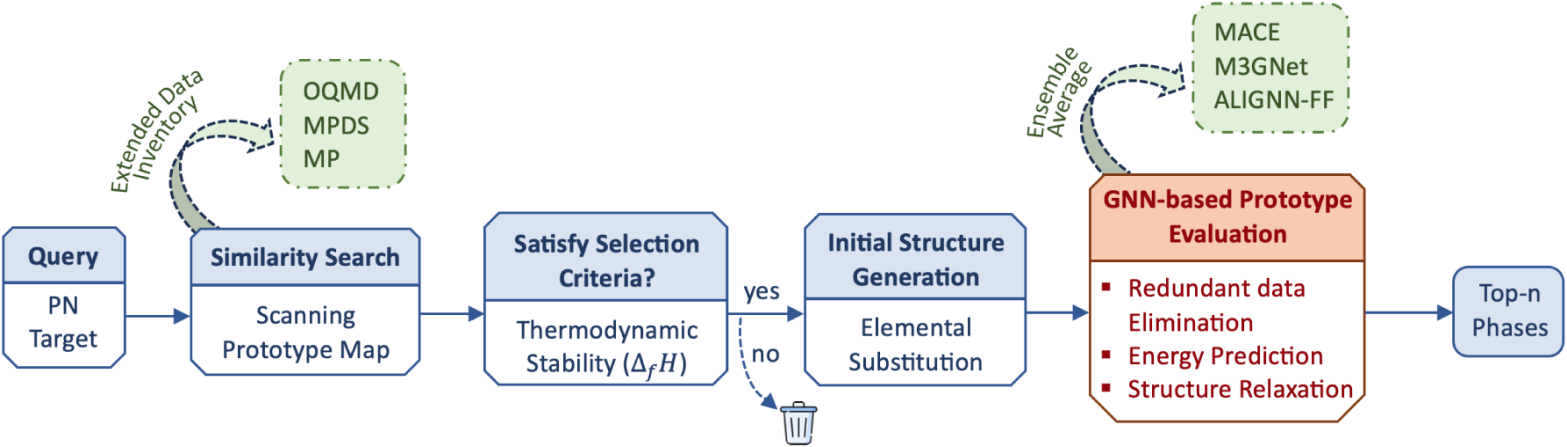
Workflow of prototype prediction with the PNcsp+ framework.

## Benchmark Set and Evaluation

### Performance Evaluation of the PNcsp+ Approach

To provide a rigorous assessment of PNcsp+, we conducted evaluations using the benchmark set, CSPBench^7^ comprising 180 crystal structures. This data set, derived from the MP database, was specifically designed to challenge CSP algorithms across a wide spectrum of difficulties. The difficulty classification accounts for multiple factors, including space group diversity, template-based categories, and elemental composition that characterize distinct crystal families. Such a design ensures that the benchmark not only captures straightforward cases but also includes complex systems where structural prediction is inherently more demanding.

To assess the predictive performance of PNcsp+ on the 180 test systems in comparison with other programs, the evaluation was carried out using two complementary criteria, following the approach of Wei et al.:^8^ (i) the success rate in reproducing the correct space group, which reflects how accurately the algorithm captures crystallographic symmetry, and (ii) a crystal structure similarity analysis, which quantifies the degree of correspondence between predicted configurations and their reference structures. Crystal structural similarity was evaluated using the StructureMatcher class from the Pymatgen library^27^ employing its default tolerance parameters (ltol = 0.2, stol = 0.3, angle_tol = 5).

### Performance of GNN Models under PNcsp+

Given the broad scope of the benchmark set, OQMD was employed as the sole data source during the evaluation. To maintain computational tractability, the nearest neighbor order was restricted to the fourth order for each constituent element, thereby effectively constraining the search radius in the phase map. If no suitable candidates were identified within the fourth order, the search was extended up to the sixth order (a condition required only for a small number of systems). This controlled neighborhood enlargement ensures that the algorithm balances chemical diversity with chemical property similarities while still capturing a sufficiently rich set of substitutional candidates.

Prototype evaluation was carried out through single-point energy predictions without structure relaxation, employing both individual GNN models and their ensemble average. Structure relaxation was intentionally excluded from the CSPBench benchmarking to emphasize a key strength of the PNcsp+ framework: the template-based search generates high-quality initial structures that are already very close to their optimized geometries. This allows reliable ranking of candidate structures using single-point energies alone. In addition, for large-scale screening tasks, such as CSPBench (180 systems spanning varying levels of structural complexity), omitting structure relaxation yields substantial computational savings while preserving competitive predictive accuracy.

Table 1 summarizes the percentage of cases in which PNcsp+ successfully identified the target structure within the top-n predictions, when evaluated using different GNN models as scoring functions. Performance of the models was compared using three evaluation metrics: (i) Space Group matching (SG), (ii) StructureMatcher matching (SM), and (iii) simultaneous agreement in both Space Group and StructureMatcher metrics (Both). Among the individual models tested, MACE (MPA-0 middle) consistently outperformed the others in top-3 and top-5 categories, while M3GNet exhibited lower but still comparable performance in these 2 cases. In top-10 predictions, M3GNet shows slightly better performance than MACE in metrics based on structure similarity, whereas both models achieve the same matching rates in the Space Group metric. On the other hand, ALIGNN-FF performed significantly worse, ranking as the last across all metrics. These observations are consistent with the mean absolute errors obtained in predicting DFT reference total energies for the CSPBench data set, as reported by the Materials Project, with values of 0.185 eV/atom for MACE, 0.194 eV/atom for M3GNet, and 2.650 eV/atom for ALIGNN-FF. Moreover, we observed that incorporating ALIGNN-FF into the ensemble averaging did not enhance the overall ranking quality. Based on these findings, the ensemble strategy was constructed by averaging the single-point energy predictions from the two best-performing models, MACE and M3GNet. Under this configuration, the ensemble model yielded the highest accuracy in the top-5 category and the second-highest performance across the remaining categories.

**Table 1:** Prediction Performance Comparison of PNcsp+ and Its Variants Using Four Nearest Neighbors^a^

	TOP-10			TOP-5			TOP-3		
PNcsp+ Model	SG	SM	Both	SG	SM	Both	SG	SM	Both
Ensemble^b^	**90.56**	86.11	85.56	**86.11**	**85.00**	**82.22**	82.22	82.78	78.89
**MACE**	**90.56**	86.11	85.56	85.56	**85.00**	81.67	**83.33**	**83.33**	**79.44**
**M3GNet**	**90.56**	**86.67**	**86.11**	84.44	83.33	81.11	78.33	80.56	75.56
**ALIGNN-FF**	87.78	80.00	78.33	76.11	72.78	70.00	62.78	64.44	59.44

a Numerical values are reported as percentages. “SG” refers to matches based on space group symmetry, “SM” to matches determined by StructureMatcher, and “Both” to predictions meeting both criteria.

b The ensemble evaluation is based on the single-point energy predictions of M3GNet and MACE models.

In Table 1, the accuracy of PNcsp+ is assessed based on its top-3, top-5, and top-10 predictions, with consideration given to the characteristics of the data set. Overall, 20% of the test structures in the data set belong to the polymorph category, consisting of structures that do not always correspond to the lowest-energy (i.e., thermodynamically most stable) configurations reported in the MP database. Consequently, evaluating PNcsp+ solely on the basis of its top-1 prediction would not provide a fair measure of performance, since in these categories the target structures may not correspond to the ground-state configurations. To enable a more rigorous evaluation of top-1 prediction accuracy, the polymorph category was extended by incorporating additional polymorphs reported in the MP database, and prediction performance was examined for both the original test set and the extended data set. Notably, inspection of the results revealed that the first-ranked predictions produced by PNcsp+ frequently corresponded to ground-state configurations, even within these challenging categories. Using the best-performing model, MACE, PNcsp+ achieved SG: 62.78%, SM: 73.89%, and Both: 61.67% on the original data set, while the performance improved to SG: 71.11%, SM: 81.67%, and Both: 69.44% on the extended data set. In contrast, M3GNet and ALIGNN-FF exhibited significantly lower performance (further details are provided in the SI).

With these settings, PNcsp+ is capable of completing the structural screening on a standard server equipped with a 32-core AMD processor in approximately ∼3 h (1 min per system on average). Based on our systematic tests, the use of four nearest neighbors provides an optimal balance between accuracy and efficiency, while increasing the order beyond this threshold yields no significant improvements in predictive accuracy. As illustrated in Table 2, the screening time for the benchmark set of 180 systems increases from ∼1.5 h when considering two neighbors to ∼3 h for four neighbors, and rises sharply to ∼15 h when the nearest neighbor order is extended to six. The number of candidate prototypes processed by the structure evaluation module strongly depends on the chemical system under consideration. Table 2 reports the average number of candidates per system across the 180 benchmark systems for different nearest neighbor settings. In contrast to other prominent template-based approaches, which typically evaluate thousands of templates^6,8,13^ PNcsp+ operates on a much smaller and more refined candidate pool, owing to its neighbor-order–guided pre-elimination strategy.

**Table 2:** Computation Time and Number of Evaluated Systems for Different Nearest Neighbor Configurations

Search Configuration	Average candidates per System	Search Time	Ensemble^a^ Evaluation Time	Total Time
2 Neighbors	78	18 min	1 h 8 min	1 h 26 min
4 Neighbors	242	23 min	2 h 42 min	3 h 5 min
6 Neighbors	677	1 h 25 min	13 h 24 min	14 h 59 min

a The ensemble evaluation is based on the single-point energy predictions of M3GNet and MACE models.

In terms of computational efficiency, M3GNet exhibited by far the highest prediction speed. MACE achieved the second-best performance but remained considerably slower than M3GNet, with prediction times approximately three times longer. This apparent performance gap, however, was partially influenced by the numerical precision used in the evaluation: M3GNet and ALIGNN were run with their default float32 data type, whereas MACE was evaluated with the recommended float64 precision. When the precision of MACE was reduced to float32, its computational speed improved noticeably, resulting in an approximately 40–50% reduction in runtime. ALIGNN-FF, meanwhile, showed the lowest computational efficiency, operating about four times slower than M3GNet.

In light of these observations, it can be concluded that, for performance-critical large-scale screening tasks, replacing the ensemble strategy with a single high-performing model such as M3GNet can significantly reduce the runtime of the prototype evaluation stage. In addition, it is worth noting that, in this performance benchmark, the GNN-based evaluation module was initialized from scratch for each individual system. In practical large-scale structure screening, deploying the evaluation module once and reusing it across the entire target system pool would provide additional performance gains.

The efficiency of PNcsp+ arises from its targeted screening strategy, which selectively yields prototypes exhibiting the most compatible structural characteristics—such as lattice parameters, bonding types, and interatomic distances—while ensuring thermodynamic stability. Low-quality candidates are systematically filtered out prior to the atomic substitution stage, thereby avoiding unnecessary computational overhead. In the subsequent step, the structure evaluation module further helps to refine the candidate pool and rapidly identify the most promising prototypes. This hierarchical selection ensures that only the most physically and chemically plausible structures advance to the final stage. Owing to this multilevel filtering, the need for the subsequent geometry optimization (either DFT- or ML-based) is significantly reduced. Remarkably, the predicted structures closely matched their experimentally determined counterparts even in the absence of structure relaxation, highlighting the intrinsic accuracy and computational efficiency of the PNcsp+ framework.

### Comparison with Different CSP Algorithms

In line with the recent comparative study by Wei et al.^8^ which evaluated the performance of their template-based program TCSP 2.0 against two leading alternatives, CSPML and EquiCSP, we adopted their published results as reference data to enable a direct and transparent comparison with our own method, PNcsp+. Notably, while the competing methods employed CHGNet-based structure relaxation, our evaluation relies exclusively on the unrelaxed structures generated by PNcsp+, ranked via an ensemble of single-point energy predictions from M3GNet and MACE.

Figure 2 compares the performance of the CSP algorithms for top-5 predictions using the same three evaluation metrics as in Table 1. PNcsp+ (Ensemble) attains the highest accuracy, achieving 86.1% in space group matching, 85.0% in structure matching, and 82.2% in the combined criterion. It outperforms its closest competitor, TCSP 2.0, by approximately 7 percentage points in structure matching (TCSP 2.0 achieves 83.9%, 78.3%, and 75.0% for the three metrics, respectively). In contrast, EquiCSP and CSPML perform considerably lower, indicating larger deviations from the target structures. The small difference between the blue and red bars for PNcsp+ suggests strong agreement not only in symmetry but also in full atomic structure. This comparison demonstrates that PNcsp+ outperforms alternative CSP frameworks in both symmetry-based and atomic-level structure matching. For top-1 predictions on the original data set, PNcsp+ outperforms the leading competitor, TCSP 2.0, in the structure matching metric (SM_PNcsp+_: 73.9%, SM_TCSP 2.0_: 68.3%). However, TCSP 2.0 achieves higher performance in the other two evaluation metrics (SG_PNcsp+_: 62.8%, SG_TCSP 2.0_: 70.6% and Both_PNcsp+_: 61.4%, Both_TCSP 2.0_: 61.7%).

**Figure 2:**
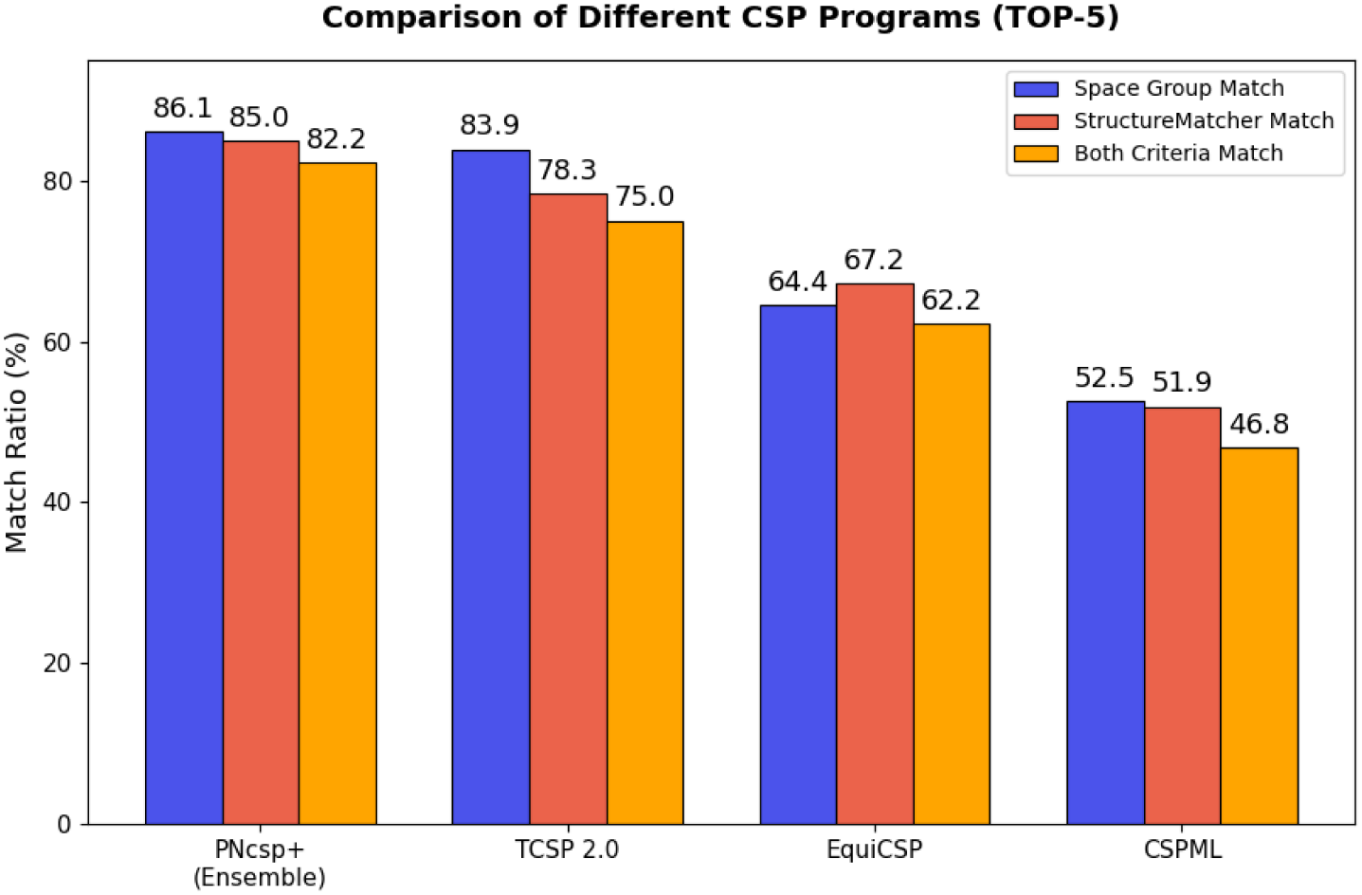
Performance comparison of PNcsp+ and other CSP approaches on the CSPbench set within the Top-5 category.

Across the full benchmark, PNcsp+ failed to identify any viable prototype candidates for five systems—Co_4_NiSb_12_, Fe_2_Cu_6_SnS_8_, MgV_4_SnO_12_, YbH_3_CN_3_, and Tb_4_Al—for distinct reasons. Regarding Co_4_NiSb_12_, the reported composition appears to be inconsistent: the phase may contain three, rather than four, formula units. Furthermore, inspection of the phase diagrams available in the Linus Pauling File (LPF) database (MPDS is the primary online access point and platform for LPF), particularly the isothermal sections at 813 and 873 K, indicates that no ternary compounds form in this system.^28^ The ternary phase listed in CSPBench is therefore likely a solid solution based on the binary CoSb_3_ phase (cubic modification) alloyed with Co–Ni, rather than a genuine ternary compound.

For MgV_4_SnO_12_ and Tb_4_Al, neither structural information nor constitution data are available in the LPF database. In the case of Tb–Al, only binary aluminide phases of rare-earth elements are documented in the Al-rich region—such as the trialuminides of Ho or Dy—suggesting that the ternary or more complex phases listed in CSPBench may not be experimentally confirmed.^29^

Interestingly, Fe_2_Cu_6_SnS_8_ and YbH_3_CN_3_ are reported in the LPF database—the former crystallizing in a tetragonal phase^30^ and the latter in a hexagonal structure belonging to the family of rare-earth metal guanidinates, containing the functional group [C–N_3_–H_3_]^2–^.^29^ However, because both compounds reside in sparsely populated regions of the OQMD-derived phase map, PNcsp+ was unable to retrieve or match suitable prototype candidates, despite the availability of corresponding structural information in LPF. This underscores the value of incorporating LPF into PNcsp+’s data inventory, as targeted searches over a small set of systems can enhance prototype-matching accuracy while adding only minimal search overhead.

Among the structures that the StructureMatcher classified as “not similar”, several cases lie near the similarity threshold. For example, KCuCl_3_ and K_3_MnO_4_ exhibit borderline average root-mean-square (RMS) distances, yet upon local relaxation using the MACE potential, their predicted structures evolve into forms that closely match the target structures. PNcsp+ also identified alternative polymorphs for some systems. As an example, LuSeO_3_F has three known polymorphs (two of which are reported as experimental structures in the MP database), and although PNcsp+ did not recover the specific target structure in the benchmark set, it successfully predicted the remaining two polymorphs. Among the experimentally reported MP polymorphs, two are monoclinic (*P*12_1_/*m*1 and *P*12_1_/*c*1) and one is triclinic (*P*1̅). Although PNcsp+ did not explicitly recover the ground-state *P*12_1_/*m*1 phase, it successfully predicted the other two polymorphs. The reported formation energies are nearly degenerate, with only ∼0.002 eV/atom separating the triclinic and ground-state structures. Structural analysis shows that the triclinic phase represents a slightly distorted variant of the ground state, consistent with the distortion pattern obtained by PNcsp+. Thus, the predicted structures remain very close to the energetic and structural ground-state landscape despite the absence of an exact match. This behavior highlights PNcsp+’s ability to navigate physically meaningful structural variants even when the exact target prototype is not captured.

### A Case Study beyond Inorganic Systems

In this section, we applied PNcsp+ to a set of structurally and chemically diverse systems—ranging from hybrid organic–inorganic perovskites (HOIPs) with halides to ammonium and antiperovskite salts—that represent some of the most challenging classes of ionic and hybrid materials. CSP for these kinds of materials is challenging due to the coexistence of ionic, covalent, and hydrogen-bonding interactions, along with orientational disorder of molecular or polyatomic cations. In addition, there is limited information available about them in the structure databases. Therefore, these systems serve as ideal test cases to evaluate PNcsp+’s capability in accurately predicting complex lattice architectures governed by competing interactions and compositional flexibility. To this end, we focused on the representative compounds including perovskite and antiperovskite structures: methylammonium lead iodide, CH_3_NH_3_PbI_3_ and some of the common precursor methylammonium halides, CH_3_NH_3_X (MAX), X = Cl, Br, I,^31–33^ as well as ammonium lead iodide, NH_4_PbI_3_; ammonium potassium sulfate, NH_4_KSO_4_, and sodium-rich chlorosulfate, Na_3_SO_4_Cl.

These compounds collectively exemplify a broad class of ionic and hybrid crystals with exceptional functional properties. Their significance arises from their diverse physicochemical behavior. For instance, HOIPs with halides have revolutionized the field of photovoltaics due to their strong light absorption and long carrier diffusion lengths^34–39^ while ammonium and alkali-metal salts are widely used for their functional properties in areas such as agriculture (fertilizers), energy storage (batteries), food processing (additives and preservatives), and as raw materials for the chemical industry (glass, soap, and bulk chemicals).^40–42^ Sodium-rich antiperovskites, in particular, are considered promising solid electrolytes for all-solid-state Na-ion batteries because of their intrinsic fast-ion mobility and structural stability under varying chemical environments.^43^

PNcsp+ successfully proposed multiple polymorphic phases for all these systems through a fully template-based strategy, without manually defined rules to specify which atoms constitute molecular subunits in hybrid systems. Given the structural complexity and the strong dependence of stability on the atomic configuration, all predicted candidates were further refined via DFT-based geometry optimization, and their enthalpies of formation were computed to assess thermodynamic stability. Remarkably, all predicted structures were found to be thermodynamically stable as shown in Table 3. In DFT calculations, CASTEP code with the generalized gradient approximation (GGA) of the exchange-correlation functional of the PBEsol type^44^ and on-the-fly generated pseudopotentials, the 80.otfg type^45^ were employed. Figures 3–5 show the corresponding optimized crystal structures. Notably, the results demonstrate that both ammonium and methylammonium molecular cations are constructed within the predicted frameworks solely based on the PN neighborhood relationships, without any explicit predefined molecular templates. The perovskite frameworks are also reproduced, as clearly observed in the cases of CH_3_NH_3_PbI_3_ and (NH_4_PbI_3_, where the methylammonium and ammonium cations occupy the cavities between the corner-sharing octahedra formed by Pb–I network (Figure 3).

**Table 3:** Calculated Enthalpies of Formation for the Selected Systems

Chemical System	Space Group	Δ_*f*_ *H* [eV/atom]
CH_3_NH_3_I	*P*2_1_/*m*	–0.440
CH_3_NH_3_I	*R*3*m*	–0.438
CH_3_NH_3_I	*Pbcm*	–0.434
CH_3_NH_3_Cl	*R*3*m*	–0.519
CH_3_NH_3_Cl	*P*2_1_/*m*	–0.514
CH_3_NH_3_Cl	*Pbcm*	–0.505
CH_3_NH_3_Br	*R*3*m*	–0.488
CH_3_NH_3_Br	*P*2_1_/*m*	–0.487
CH_3_NH_3_Br	*Pbcm*	–0.478
CH_3_NH_3_PbI_3_	*Pnma*	–0.541
CH_3_NH_3_PbI_3_	*Pm*	–0.535
NH_4_PbI_3_	P2_1_	–0.684
NH_4_PbI_3_	R3	–0.661
NH_4_KSO_4_	*P*2_1_/*c*	–1.364
NH_4_KSO_4_	Pna2_1_	–1.363
NH_4_KSO_4_	*Pnma*	–1.363
NH_4_KSO_4_	Pca2_1_	–1.352
Na_3_SClO_4_	*P*4/*nmm*	–1.965
Na_3_SClO_4_	*R*3*m*	–1.922
Na_3_SClO_4_	P4̅3m	–1.922
Na_3_SClO_4_	*I*4/*mcm*	–1.900

**Figure 3:**
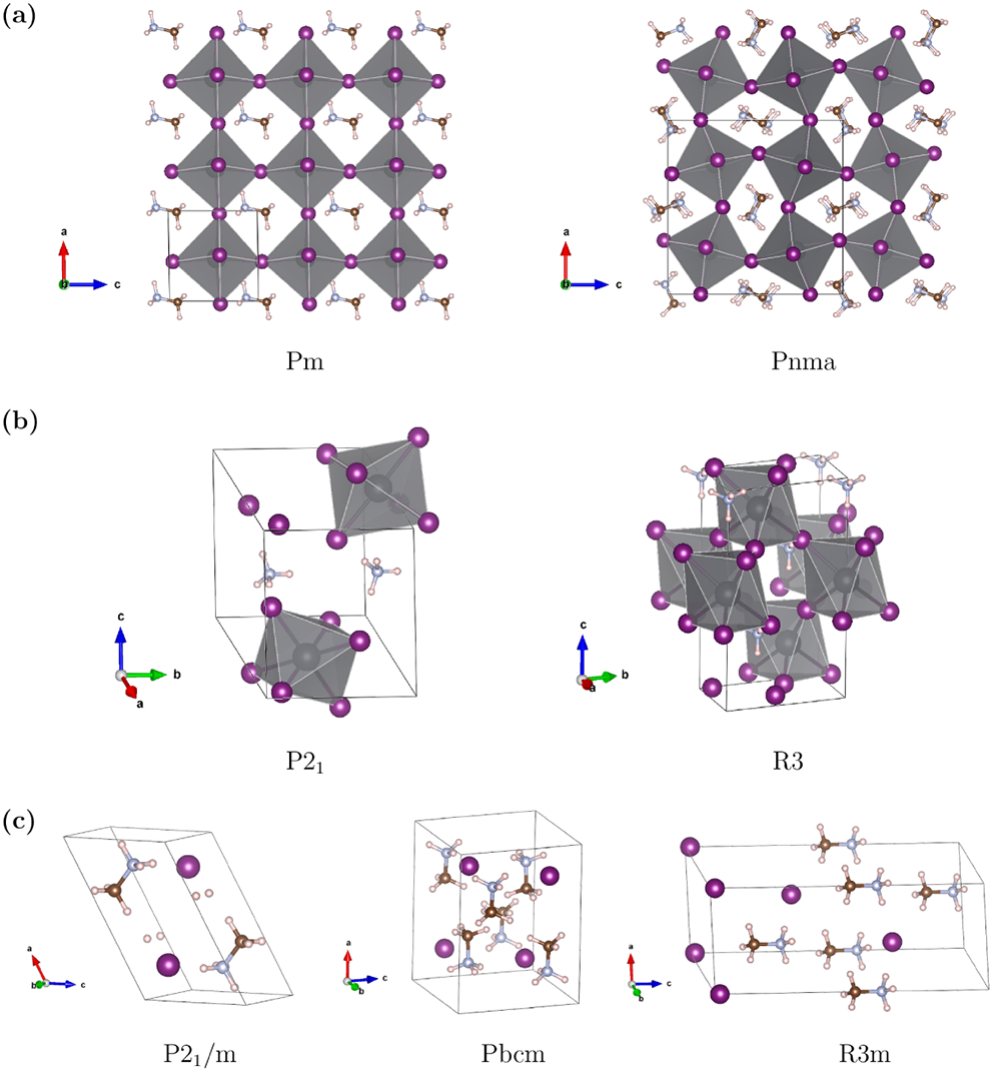
Optimized structures of CH_3_NH_3_PbI_3_ (a) and NH_4_PbI_3_ (b) perovskites and CH_3_NH_3_I (c) salt representing the typical lattice arrangement of methylammonium halides. PbI_6_ octahedra are highlighted in gray within corresponding structures.

Among these systems, CH_3_NH_3_PbI_3_ has attracted the most extensive attention owing to its technological and industrial importance. In the literature up to date, three modifications are reported for CH_3_NH_3_PbI_3_: the cubic phase stable above room temperature, the tetragonal phase stable around room temperature and below, and a low-temperature phase with orthorhombic structure.^46,47^ By using our methodology described in the present work, we found two possible stable modifications of CH_3_NH_3_PbI_3_ with monoclinic and orthorhombic symmetry representations, being the latter slightly lower in energy than the former, as reported in Table 3. The monoclinic modification with Pearson’s symbol mP12 and space group number 6 can be interpreted as a distorted perovskite structure, where the center of mass of the methylammonium cations sits on the corner of a distorted cubic lattice, the (1a) position of the ideal cubic perovskite structure, and Pb atoms occupy the body-centered position of the lattice. Similarly, the orthorhombic modification (oP48,62) is a variation of the orthorhombic perovskite phase with prototype GdFeO_3_, oP20,62 adopted by CaTiO_3_ in the room-temperature phase.

Notably, PNcsp+ successfully identified structures that closely match those described in previous studies.^48–50^ Regarding the precursor salts, the MP database reports two structures for CH_3_NH_3_I with *Pbcm* ^51^ and *P*2_1_ ^52^ space groups, while CH_3_NH_3_Cl has a single entry with *Pbcm* symmetry^51^ and no entry is available for CH_3_NH_3_Br (similarly, no entries exist for these systems in OQMD). Remarkably, PNcsp+ not only recovered these reported structures but also predicted an additional polymorph with *R*3*m* symmetry, which exhibits the lowest enthalpy of formation among the CH_3_NH_3_Cl and CH_3_NH_3_Br systems.

The rhombohedral phase of methylammonium halides derived from the low-temperature (123 K) structure of methylammonium fluoride, initially determined experimentally^53,54^ and later examined computationally for phase stability.^55^

For NH_4_PbI_3_, experimental reports indicate that the compound primarily adopts an orthorhombic crystal structure with *Pnma* symmetry^56^ while the MP database lists an additional *Cm* polymorph.^51,52^ The PNcsp+ identified two competitive alternatives—a monoclinic (*P*2_1_) and a trigonal phase (*R*3)—whose enthalpies of formation are comparable to that of the experimentally observed orthorhombic phase, reported as −0.675 eV per atom.

Regarding the crystal structure of NH_4_KSO_4_, two orthorhombic phases have been reported: one with *Pnma* symmetry and a slightly modified variant with *Pna*2_1_ symmetry, as indicated in two previous studies.^57,58^ The PNcsp+ not only reproduced these reported structures but also predicted two additional modifications with *P*2_1_/*c* and *Pca*2_1_ symmetries, the former exhibiting a slightly lower enthalpy of formation than the others. As shown in Figure 4, SO_4_ tetrahedra coordinate with potassium atoms via oxygen atoms, forming the fundamental inorganic framework. The ammonium ions are linked to this framework through an extensive network of hydrogen bonds. In the lower two structures, exhibiting *Pnma* and *P*2_1_ symmetries, the crystal structure adopts a compact and highly interconnected topology. In contrast, the *P*2_1_/*c* and *Pca*2_1_ variants display a more open framework, where the enlarged voids accommodate the ammonium cations within the resulting cavities.

**Figure 4:**
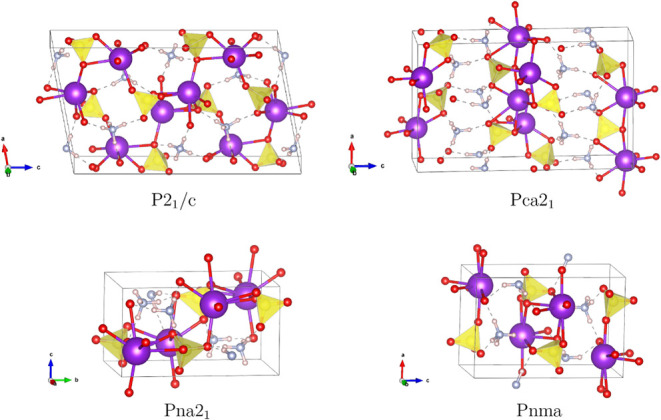
Optimized structures of NH_4_KSO_4_. SO_4_ tetrahedra are highlighted in yellow.

For Na_3_SClO_4_, although an experimental study has confirmed the formation of the compound based on the X-ray diffraction patterns^59^ no direct structural information has been reported. A prior theoretical study^60^ proposed a hypothetical cubic structure with *Fm*3*®m* symmetry and suggested its stability. PNcsp+, however, uncovered four distinct structural candidates: cubic (*P*4̅3*m*), tetragonal (*P*4/*nmm* and *I*4/*mcm*), and trigonal (*R*3*m*) phases, with the *P*4/*nmm* configuration exhibiting the lowest enthalpy of formation (Table 3). It is worth noting that the *Fm*3̅*m* structure proposed in the study of Xu et al.^60^ possesses a significantly higher enthalpy of formation compared to PNcsp+’s outcomes. Figure 5 shows that two types of polyhedra interconnect to stabilize the lattice: the smaller polyhedra formed by sulfur and oxygen are encapsulated within a framework composed of chlorine and sodium (located interior), yielding a balanced and cohesive structure.

**Figure 5:**
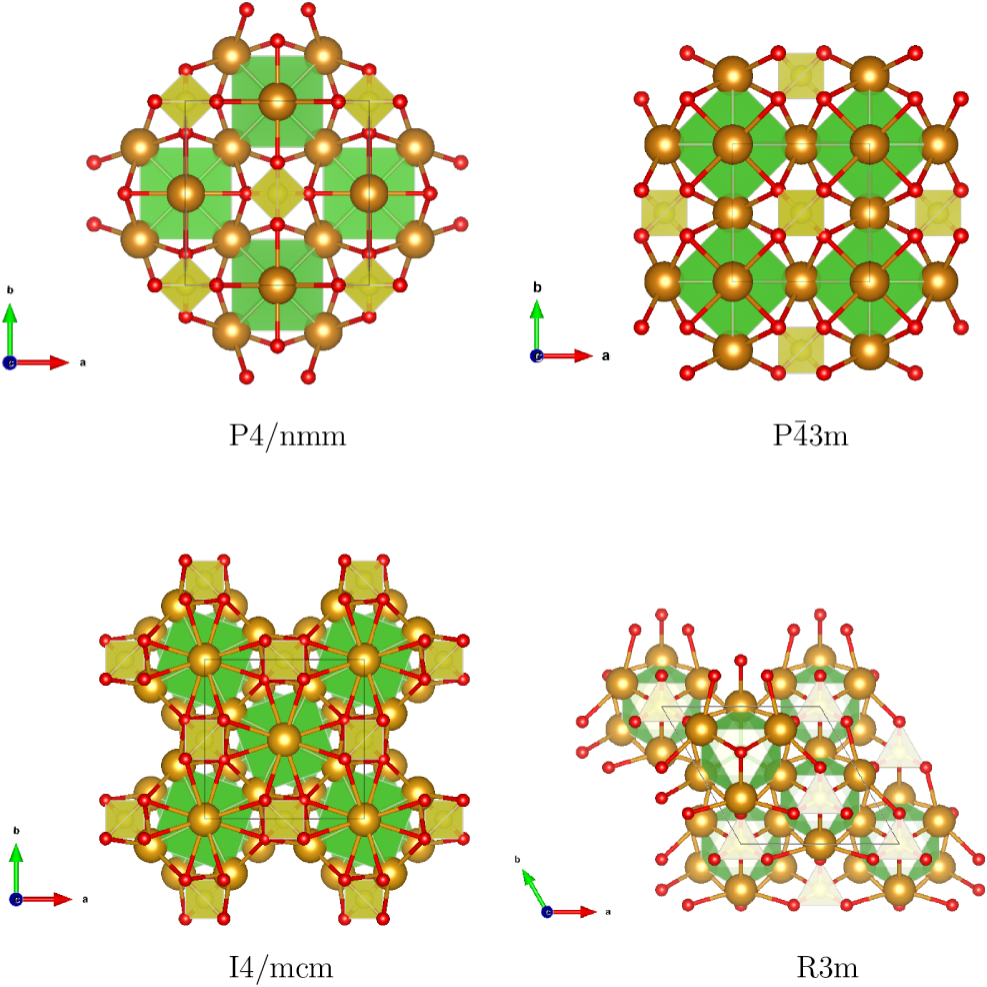
Optimized perovskite structures of Na_3_SClO_4_. SO_4_ tetrahedra are highlighted in yellow, and the ClNa_3_ octahedra are highlighted in green, with Na atoms depicted in orange.

## Conclusion

PNcsp+ offers a chemically interpretable and computationally efficient route for CSP by harnessing Mendeleev PN to quantify elemental similarity. Its hierarchical screening across multiple databases, coupled with a prototype evaluation pipeline that integrates diverse GNN models, enables the systematic identification of structurally consistent and thermodynamically stable candidates within a minimal computational overhead.

Comprehensive benchmarking against established CSP approaches reveals that PNcsp+ surpasses existing methods in identifying structurally and energetically plausible prototypes for inorganic systems with a diverse range of complexity. PNcsp+ with the ensemble averaging scheme achieved its best performance, attaining 86.1% accuracy in space group classification and 85.0% in structure matching within the Top-5 predictions without reliance on structure relaxation. Furthermore, its successful application to hybrid organic–inorganic compounds highlights the framework’s versatility beyond purely inorganic chemistry. Applications to hybrid systems including ammonium and methylammonium compounds, remarkably demonstrate that the molecular cations emerge naturally within the predicted lattice frameworks—guided solely by PN-derived neighborhood relationships and without reliance on manually defined molecular templates—while accurately reproducing the underlying crystalline architectures. By capturing fundamental structure–chemistry relationships, PNcsp+ reveals that fundamental periodic trends can be leveraged to accelerate materials discovery while maintaining high predictive accuracy.

## Supplementary Material



## Data Availability

An open-source software implementation of our similarity-based initial crystal structure prediction method is available at https://github.com/tccdem/PNcsp_Plus.

## Notes

A detailed description of the PN concept, Top-1 performance metrics of PNcsp+, and more information about the CSP for LuSeO_3_F are provided (PDF)
