# Supplementary material for: PNcsp+: A Periodic Number-Based Crystal Structure Prediction Method Enhanced by Machine Learning
Source: J Chem Theory Comput. 2026 Mar 19;22(7):3761–71. doi: 10.1021/acs.jctc.6c00044 (PMC13085239; doi:10.1021/acs.jctc.6c00044)
Supplement: Supplementary file 1 [file ct6c00044_si_001.pdf]

Supplementary Information: PNcsp+: A Periodic Number-Based Crystal Structure Prediction Method Enhanced by Machine Learning

Cem Oran,[†] Riccarda Caputo,[‡] Pierre Villars,[¶] and Adem Tekin*[*,†,§]

†*Informatics Institute, Istanbul Technical University, 34469 Istanbul, Türkiye*

‡*Computational Materials Informatics - CMI, 00157 Rome, Italy*

¶*MPDS-Villars, 6354 Vitznau, Switzerland*

§*TÜBİTAK Research Institute for Fundamental Sciences 41470 Gebze, Türkiye*

E-mail: adem.tekin@itu.edu.tr

Mendeleev's Periodic Number (PN) Concept

The PN concept integrates two fundamental criteria: the principal quantum number, which governs the atomic energy level and defines the periods of the Periodic Table, and the valence-shell electron configuration, which determines the chemical character of the elements. For the systematic enumeration of the 118 chemical elements according to the Mendeleev Periodic Table, we introduce a methodology that starts with the principal quantum number (n) equal to 2, proceeding sequentially through the groups up to $n = 7$, thus covering all seven rows of the Periodic Table. The enumeration initiates with elements possessing a valence shell configuration of ns^1, starting from $n = 2$ to $n = 7$. As a result, the PN for lithium (Li) is assigned 1, sodium (Na) is assigned 2, and so forth, incrementally covering all alkali metals characterized by the outermost configuration ns^1.

For elements with fully occupied ns^2 shells (the earth-alkaline metals), the enumeration starts from $n = 4$ and continues to $n = 7$, including calcium (Ca) to radium (Ra). Within this framework, beryllium (Be) and magnesium (Mg) are reassigned to Group 12 of the Periodic Table. Subsequently, beginning again from $n = 4$, the available 3d, 4d (singly occupied), and f-shells (4f and 5f) are filled. Thus, PN = 11 corresponds to scandium (Sc), PN = 12 to yttrium (Y), and from PN = 13 to PN = 42 correspond to the lanthanides and actinides, characterized by their respective configurations $4f^{1-14}$ and $5f^{1-14}$.

Following the completion of the deeper f-electron subshells, filling proceeds into the outer $(n-1)d$ levels. Elements in this region exhibit valence shell configurations such as $ns^2(n-1)d^{2-3}$ (Ti and V groups), $ns^1(n-1)d^5$ (Cr group), $ns^2(n-1)d^{5-8}$ (Mn, Fe, Co, Ni groups), and $ns^1(n-1)d^{10}$ (Cu group). The enumeration resumes from $n = 2$, completing the ns^2 configurations, as seen in Be ($2s^2$) and Mg ($3s^2$). The process continues with $ns^2(n-1)d^{10}$ for $n = 4$ to $n = 7$, where the $(n-1)d$ states are fully occupied and have lower energy than the ns^2 states.

In the final stage, the enumeration proceeds by filling the np orbitals. For $n = 2$ to $n = 7$, elements display configurations such as ns^2np^1, ns^2np^2, ns^2np^3, and ns^2np^4. Additionally,

for $n = 1$ through $n = 7$, the ns^1np^6 and ns^2np^6 configurations are completed. The PN enumeration of the complete set of Periodic Table elements is listed in Table S1 and visualized in Figure S1, which presents a modified version of the figure reported in our previous work (ref. 15 in the main text).

Table S1: Mendeleev's Periodic Number (PN) enumeration for the complete set of Periodic Table elements. Z is the atomic number.

**Symbol**	Li	Na	K	Rb	Cs	Fr	Ca	Sr	Ba	Ra	Sc	Y	La	Ac	Ce	Th	Pr	Pa	Nd	U
**PN**	1	2	3	4	5	6	7	8	9	10	11	12	13	14	15	16	17	18	19	20
**Z**	3	11	19	37	55	87	20	38	56	88	21	39	57	89	58	90	59	91	60	92
**Symbol**	Pm	Np	Sm	Pu	Eu	Am	Gd	Cm	Tb	Bk	Dy	Cf	Ho	Es	Er	Fm	Tm	Md	Yb	No
**PN**	21	22	23	24	25	26	27	28	29	30	31	32	33	34	35	36	37	38	39	40
**Z**	61	93	62	94	63	95	64	96	65	97	66	98	67	99	68	100	69	101	70	102
**Symbol**	Lu	Lr	Ti	Zr	Hf	Rf	V	Nb	Ta	Db	Cr	Mo	W	Sg	Mn	Tc	Re	Bh	Fe	Ru
**PN**	41	42	43	44	45	46	47	48	49	50	51	52	53	54	55	56	57	58	59	60
**Z**	71	103	22	40	72	104	23	41	73	105	24	42	74	106	25	43	75	107	26	44
**Symbol**	Os	Hs	Co	Rh	Ir	Mt	Ni	Pd	Pt	Ds	Cu	Ag	Au	Rg	Be	Mg	Zn	Cd	Hg	Cn
**PN**	61	62	63	64	65	66	67	68	69	70	71	72	73	74	75	76	77	78	79	80
**Z**	76	108	27	45	77	109	28	46	78	110	29	47	79	111	4	12	30	48	80	112
**Symbol**	B	Al	Ga	In	Tl	Nh	C	Si	Ge	Sn	Pb	Fl	N	P	As	Sb	Bi	Mc	O	S
**PN**	81	82	83	84	85	86	87	88	89	90	91	92	93	94	95	96	97	98	99	100
**Z**	5	13	31	49	81	113	6	14	32	50	82	114	7	15	33	51	83	115	8	16
**Symbol**	Se	Te	Po	Lv	H	F	Cl	Br	I	At	Ts	He	Ne	Ar	Kr	Xe	Rn	Og		
**PN**	101	102	103	104	105	106	107	108	109	110	111	112	113	114	115	116	117	118		
**Z**	34	52	84	116	1	9	17	35	53	85	117	2	10	18	36	54	86	118		

By mapping the PNs of the constituent elements, one can systematically construct phase maps whose dimensionality directly reflects the system's compositional complexity: two-dimensional maps for binaries, three-dimensional representations for ternaries, and correspondingly higher-dimensional spaces for multicomponent systems. What sets this representation apart from earlier phase-map descriptors is its ability to explicitly delineate chemically meaningful domains: regions where compounds are observed to form and regions where no stable phases are found except for a few accepted violations. Table S2 summarizes the boundaries defining the identified forbidden regions, which are also illustrated in Figure S3. Notably, the relative fraction of non-forming systems systematically declines with increasing chemical complexity: 26.7% (1,846 systems) for binaries, 10% (26,870 systems) for ternaries, and 4.2% (324,256 systems) for quaternaries.

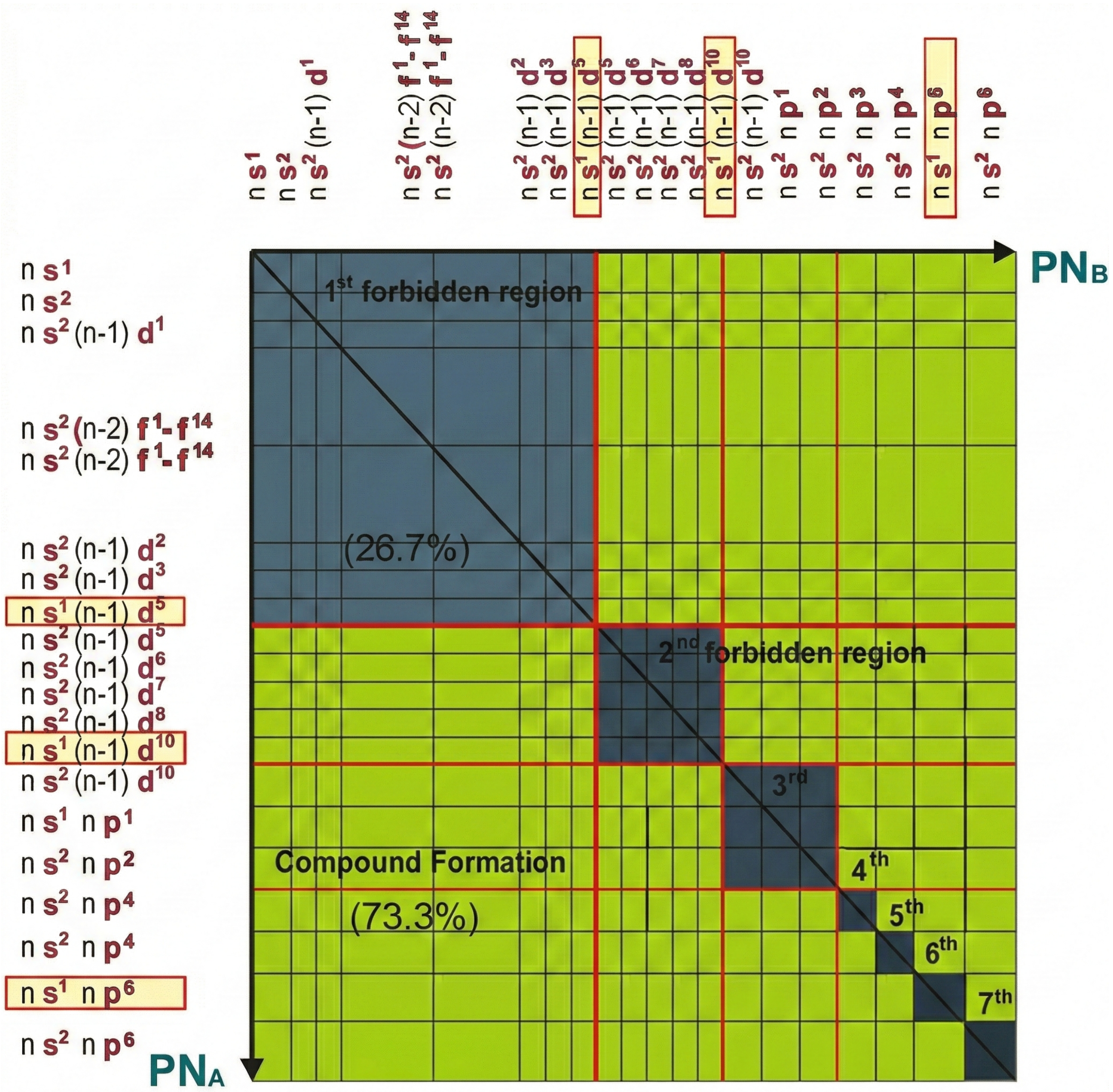

Figure S1. Binary phase map of the complete set of Periodic Table elements.

In doing so, it does not merely separate "former" and "non-former" areas, but also makes underlying chemical property trends visually and conceptually transparent. As a demonstration of the approach, we integrated crystallographic data from the LINUS PAULING FILE with first-principles calculations in our previous study, Caputo et al. (2024) (ref. 15 in the main text). This integrated analysis showed that the PN-based representation naturally captures systematic trends in key material properties of binary compounds, including enthalpy

of formation, cohesive energy, mechanical characteristics such as bulk modulus and structural prototypes. For a comprehensive description of the methodology and its implications, we refer the reader to Caputo et al. (2024), where the analysis is presented and discussed in full detail.

Table S2: Seven forbidden regions for non-former systems are identified. A system is designated as a non-former within a given region when the PNs of all constituent elements lie inside the corresponding PN interval.

Forbidden Regions				# No. chemical systems		
Region	PN range	Element span	# elements	Binary	Ternary	Quaternary
1	1–54	Li–Sg	54	1431	24804	316251
2	55–74	Mn–Rg	20	190	1140	4845
3	75–92	Be–Fl	18	153	816	3060
4	93–98	N–Mc	6	15	20	15
5	99–104	O–Lv	6	15	20	15
6	105–111	H–Ts	7	21	35	35
7	112–118	He–Og	7	21	35	35

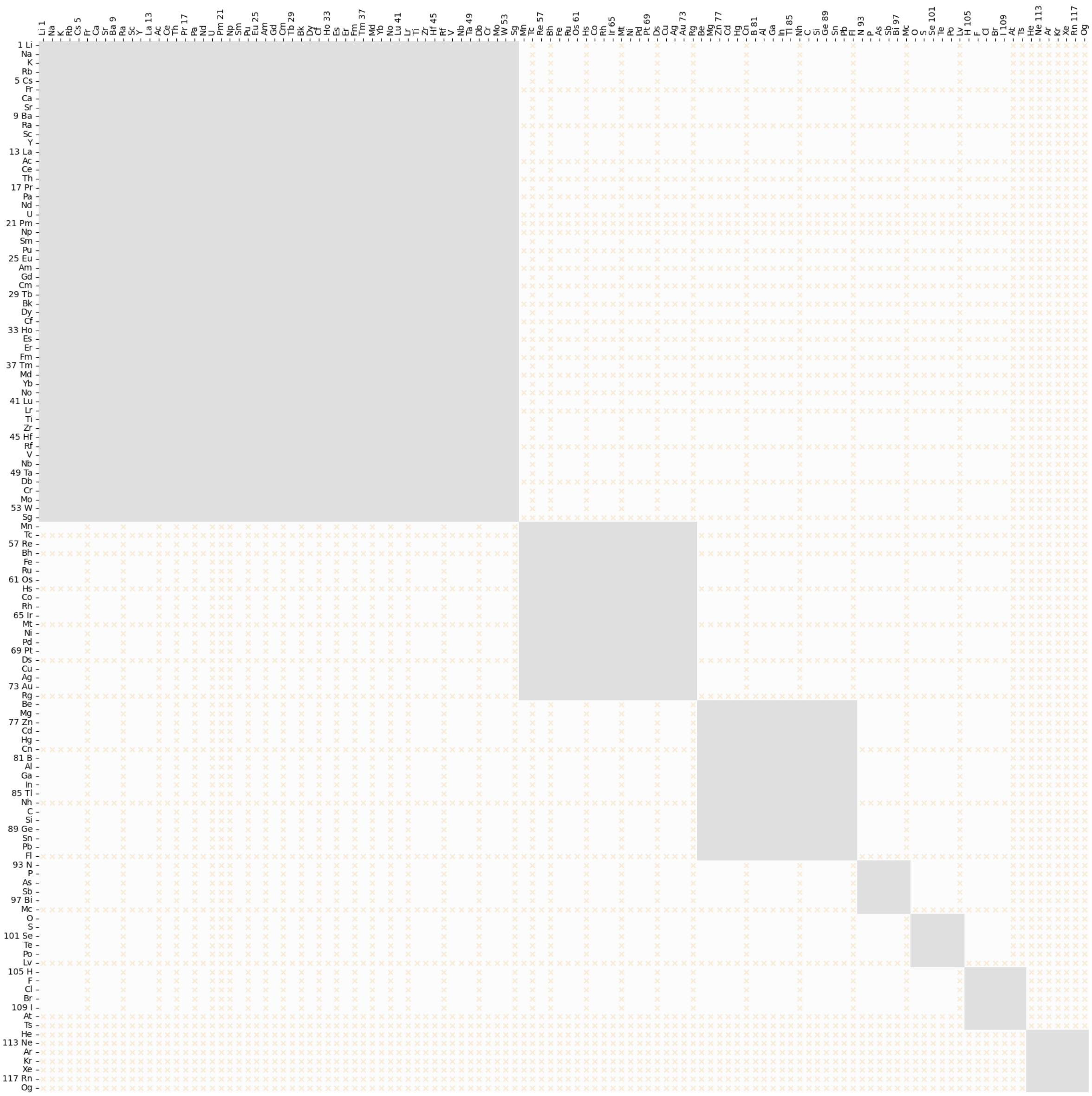

Figure S2. Seven forbidden regions are highlighted in grey in the binary phase map.

Top-1 Performance Metrics

Table S3 compares top-1 prediction performance across the extended and original datasets using three evaluation metrics: space-group match (SG), structure match (SM), and their joint criterion (Both). Overall, performance is consistently higher on the extended dataset for all models and metrics. Among the individual ML models, MACE achieves the strongest

Table S3: Top-1 performance comparison. The first set of metrics columns corresponds to the extended dataset, and the second set corresponds to the original dataset.

**PNcsp+ Model**	Extended data			Original data		
	SG	SM	Both	SG	SM	Both
Ensemble	71.11	79.44	68.89	62.22	71.67	60.56
MACE	71.11	81.67	69.44	62.78	73.89	61.67
M3GNet	62.78	72.22	61.67	53.89	63.89	53.33
ALIGNN-FF	46.11	49.44	43.89	41.67	43.89	40.00

results on the extended dataset, delivering the highest SM (81.67%) and Both (69.44%) scores, and sharing the top SG value (71.11%) with the Ensemble baseline. M3GNet shows intermediate performance, while ALIGNN-FF exhibits the lowest accuracy across all metrics.

Crystal Structure Prediction for $LuSeO_3F$

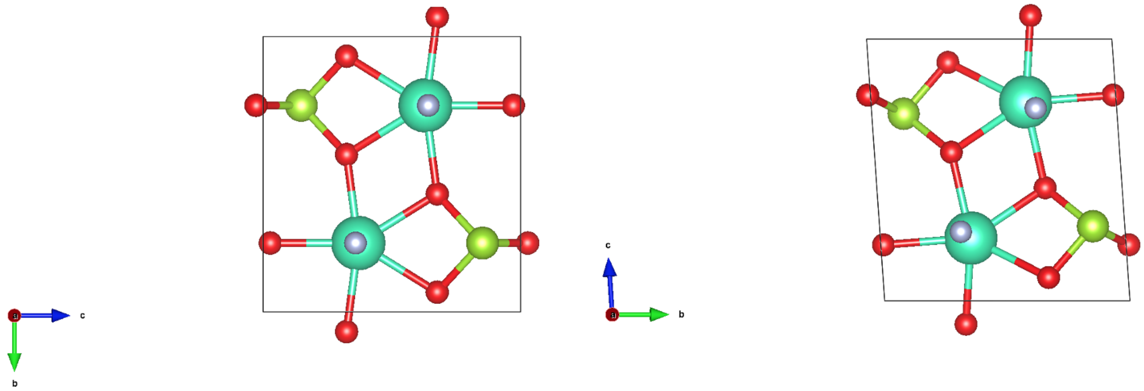

Figure S3. Left: Ground State structure ($P12_1/m1$), Right: PNcsp+'s prediction ($P\bar{1}$).

Regarding the experimentally reported polymorphs in MP, two structures are monoclinic ($P12_1/m1$ and $P12_1/c1$) and one is triclinic ($P\bar{1}$). PNcsp+ couldn't identify the ground-state $P12_1/m1$ phase explicitly but successfully predicted the other two polymorphs in terms of both symmetry and structure similarity. Importantly, the formation energies reported in MP for these structures are extremely close: the energy difference between the triclinic structure and the ground-state monoclinic structure is approximately 0.002 eV/atom (The energy difference between 2 monoclinic structures is 0.04 eV/atom).

Structural analysis indicates that the triclinic phase can be interpreted as a slightly distorted variant of the ground-state structure, and the structure predicted by PNcsp+ exhibits a closely related distortion pattern as seen in Figure S3. Therefore, although the exact ground-state symmetry was not recovered in this benchmark configuration, the predicted structures lie very close to the energetic and structural ground-state landscape.
